# Supplementary material for: Potential impacts of climate change on agriculture and fisheries production in 72 tropical coastal communities
Source: Nat Commun. 2022 Jul 5;13:3530. doi: 10.1038/s41467-022-30991-4 (PMC9256605; doi:10.1038/s41467-022-30991-4)
Supplement: Supplementary file 1 — Supplementary Information [file 41467_2022_30991_MOESM1_ESM.pdf]

Supplementary Information

Potential impacts of climate change on agriculture and fisheries production in 72 tropical coastal communities

Joshua E. Cinner*, Iain R. Caldwell, Lauric Thiault, John Ben, Julia L. Blanchard, Marta Coll, Amy Diedrich, Tyler D. Eddy, Jason D. Everett, Christian Folberth, Didier Gascuel, Jerome Guiet, Georgina G. Gurney, Ryan F. Heneghan, Jonas Jägermeyr, Narriman Jiddawi, Rachael Lahari, John Kuange, Wenfeng Liu, Olivier Maury, Christoph Müller, Camilla Novaglio, Juliano Palacios-Abrantes, Colleen M. Petrik, Ando Rabearisoa, Derek P. Tittensor, Andrew Wamukota, Richard Pollnac

*correspondence to: joshua.cinner@jcu.edu.au

CONTENTS

Supplementary Figures:

Supplementary Figure 1: Projected agricultural changes by crop for two climate mitigation scenarios: SSP1-2.6 and SSP5-8.5.

Supplementary Figure 2. A comparison of expected fisheries and agriculture losses (exposure) for two climate mitigation scenarios.

Supplementary Figure 3. Potential impacts of changes to agriculture and fisheries by climate change mitigation scenario.

Supplementary Figure 4. Relationships between material style of life and (a) agriculture-fisheries sensitivity, or (b) agriculture-fisheries exposure under two climate mitigation scenarios (SSP1-2.6 and SSP5-8.5).

Supplementary Figure 5. Cells used in determining fisheries and agriculture exposure.

Supplementary Figure 6. Trade-off between model agreement and number of cells used for fisheries SSP1-2.6.

Supplementary Figure 7. Trade-off between model agreement and number of cells used for fisheries SSP5-8.5.

Supplementary Figure 8. Spatial extent covered by using different numbers of grid cells to determine fisheries exposure.

Supplementary Figure 9. Inset map of study sites and average agriculture-fisheries model run agreement at each study site (n = 72).

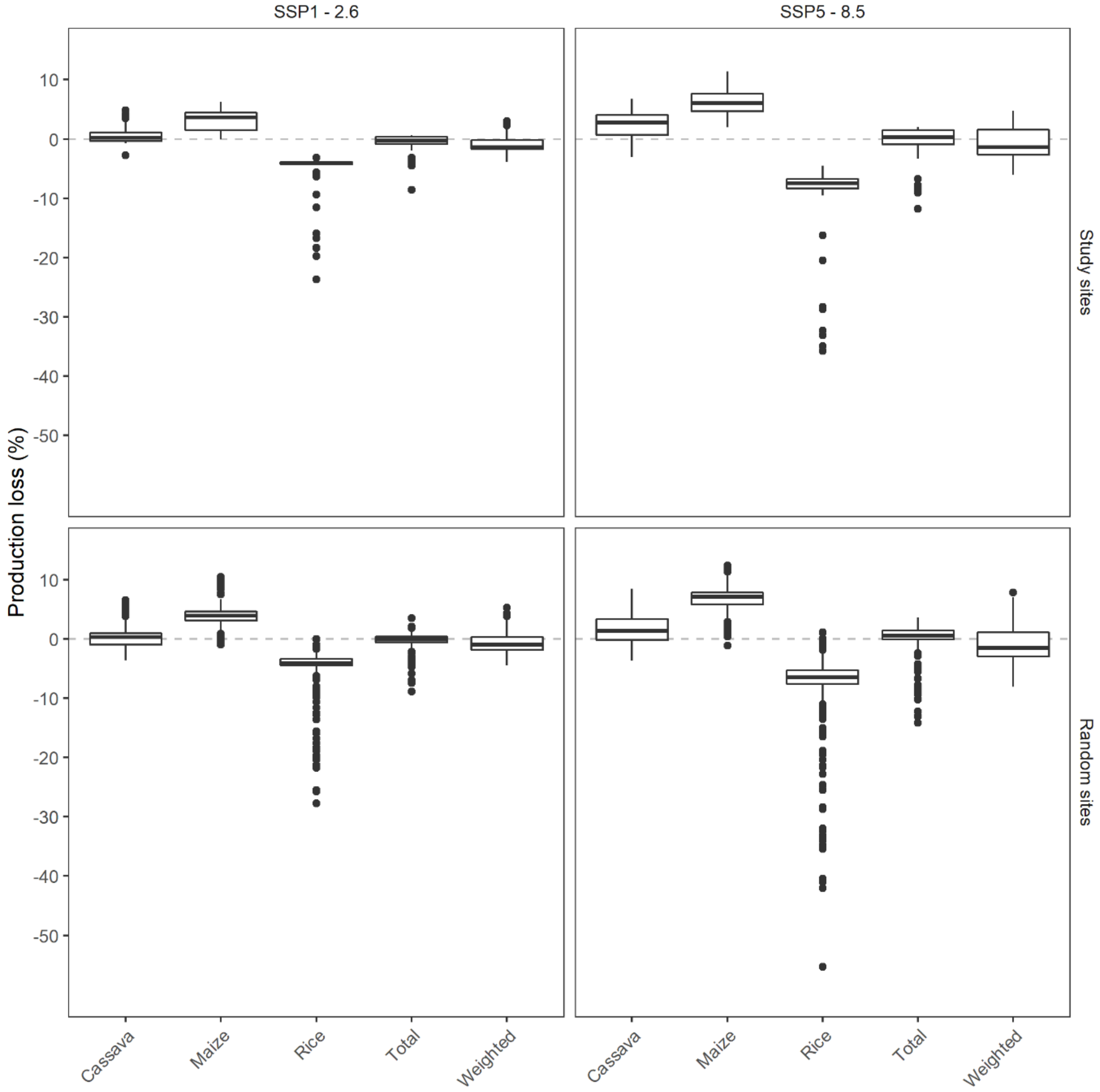

Supplementary Figure 1. **Projected agricultural changes by crop for two climate mitigation scenarios: SSP1-2.6 and SSP5-8.5**. Top row are projections for our study sites (n = 72), while the bottom row examines projected changes for a random selection of 10% of coastal sites from our study region (n = 4746). "Weighted" projections are based on agricultural projections weighted by current yields/production area of each crop. Boxplots show the median (thick line), interquartile range (hinges), minimum and maximum values within 1.5 times the interquartile range (whiskers), and any outliers beyond the whiskers (points).

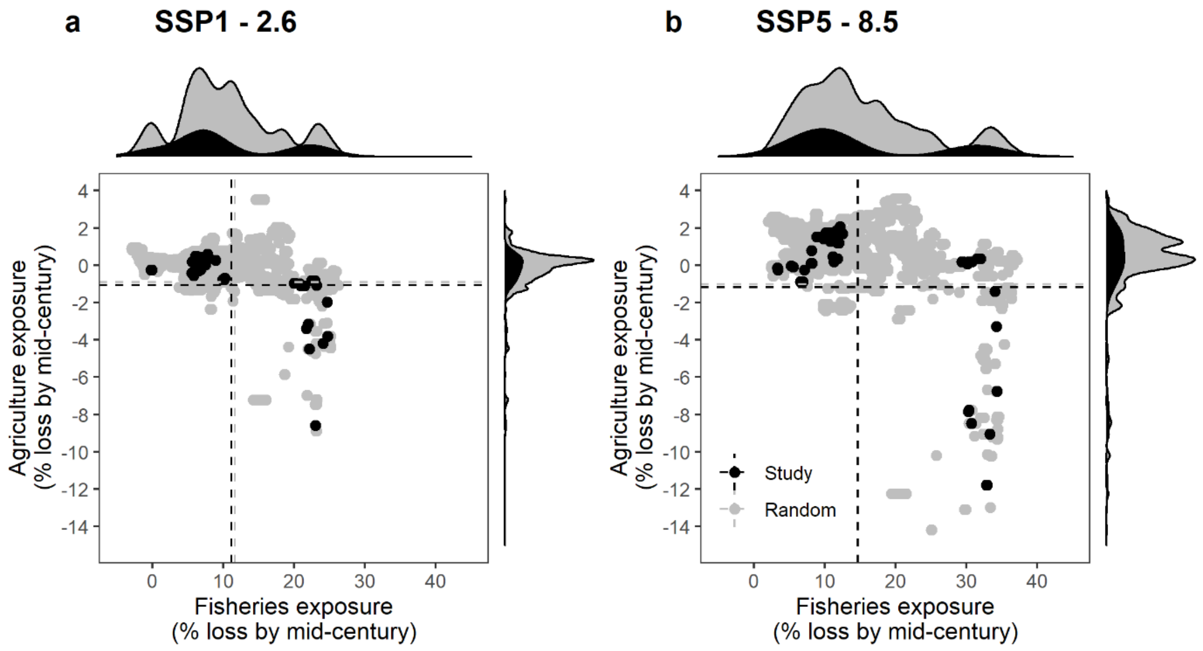

Supplementary Figure 2. **A comparison of expected fisheries and agriculture losses (exposure) for two climate mitigation scenarios: (a) SSP1-2.6 and (b) SSP5-8.5**. Black dots, histograms, and dotted lines (for mean exposures) represent our study sites (n = 72). Grey dots, histograms, and dotted lines are a random selection of 10% of coastal cells with population densities >25 people/km^2 (n = 4746). Differences between expected losses in our sites and the randomly selected sites are generally small to negligible (Cohen's D for agricultural losses SSP5-8.5 = 0.31, SSP1-2.6 = 0.35, fisheries losses SSP5-8.5 = -0.02, RCP2.6 = -0.03), indicating that our sites are not particularly biased.

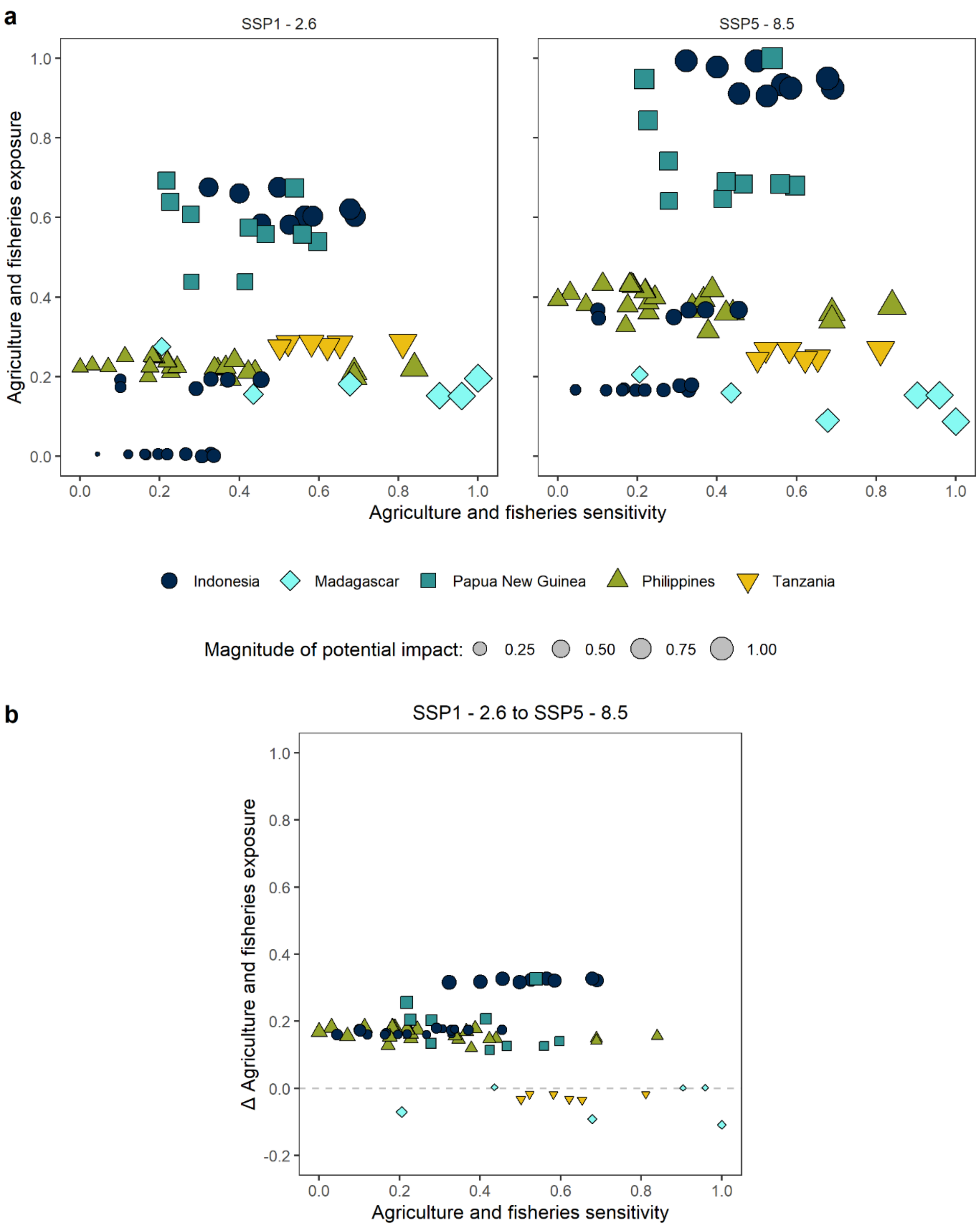

Supplementary Figure 3. **Potential impacts of changes to agriculture and fisheries by climate change mitigation scenario**. **a** Projected exposures for two mitigation scenarios (SSP1-2.6 and SSP5-8.5) compared to sensitivities. Both exposure and sensitivity to fisheries and agriculture are integrated. The potential impact is calculated as the Euclidian distance to the origin. **c** The change in potential impact from mitigation (i.e. the difference between SSP5-8.5 and SSP1-2.6). Colours and shapes indicate countries while bubble sizes indicate the magnitude of impact.

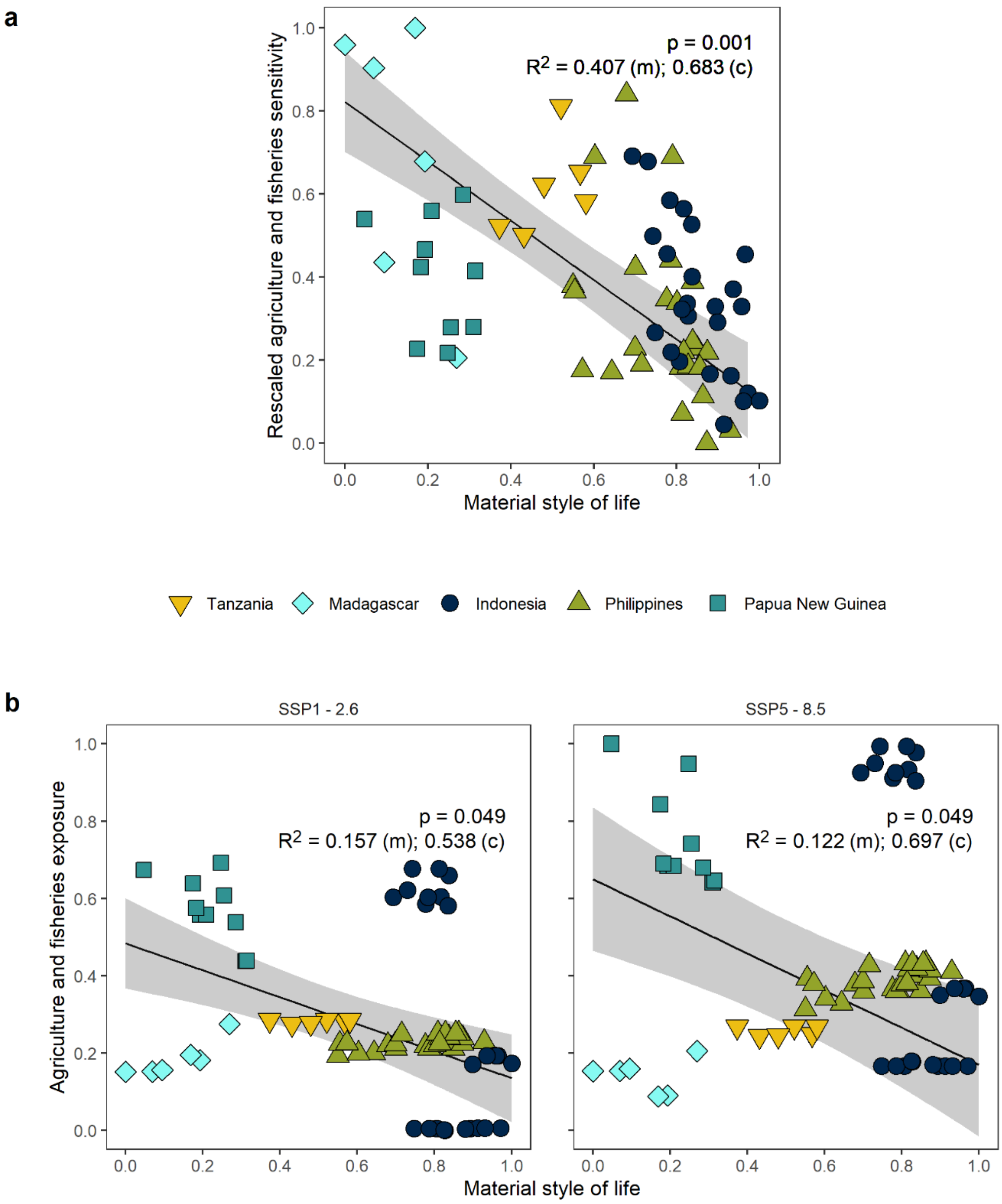

Supplementary Figure 4. **Relationships between material style of life and (a) agriculture-fisheries sensitivity, or (b) agriculture-fisheries exposure under two climate mitigation scenarios (SSP1-2.6 and SSP5-8.5) across all studied communities (n = 72)**. Black lines are predictions from linear mixed effects models (with country as random effect) and grey bands are standard errors. Statistical significance (p) and fit (R2) of the mixed effects models are also shown: (m) = marginal R^2, (c) = conditional R^2. Point colours and shapes indicate country.

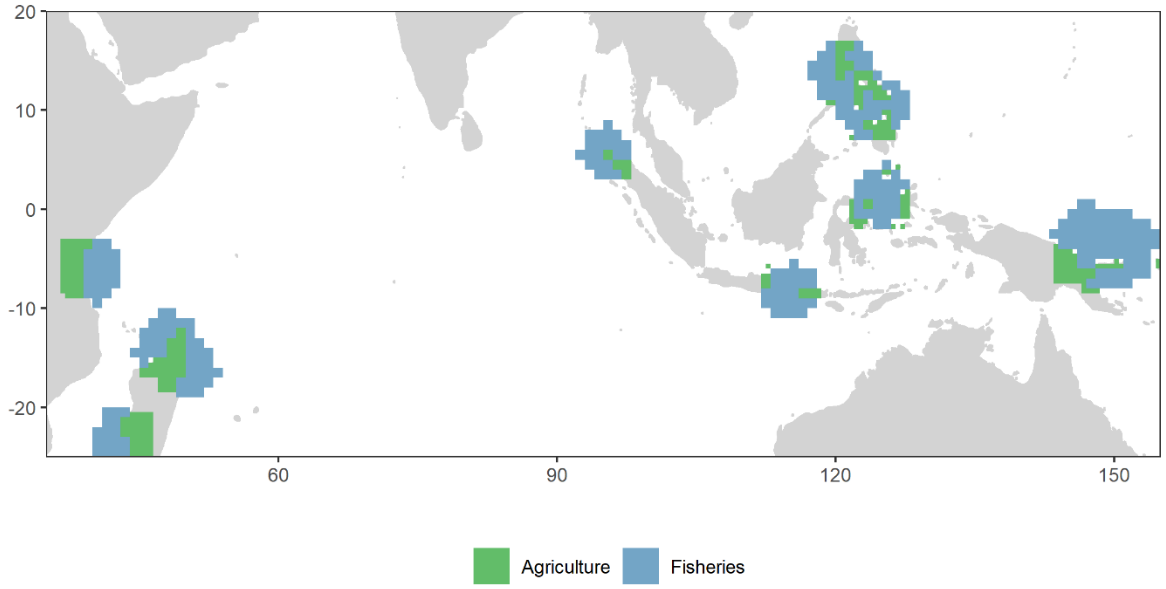

Supplementary Figure 5. **Cells used in determining agriculture (green) and fisheries (blue) exposure**. Base layer map data from Natural Earth (freely available at naturalearthdata.com)

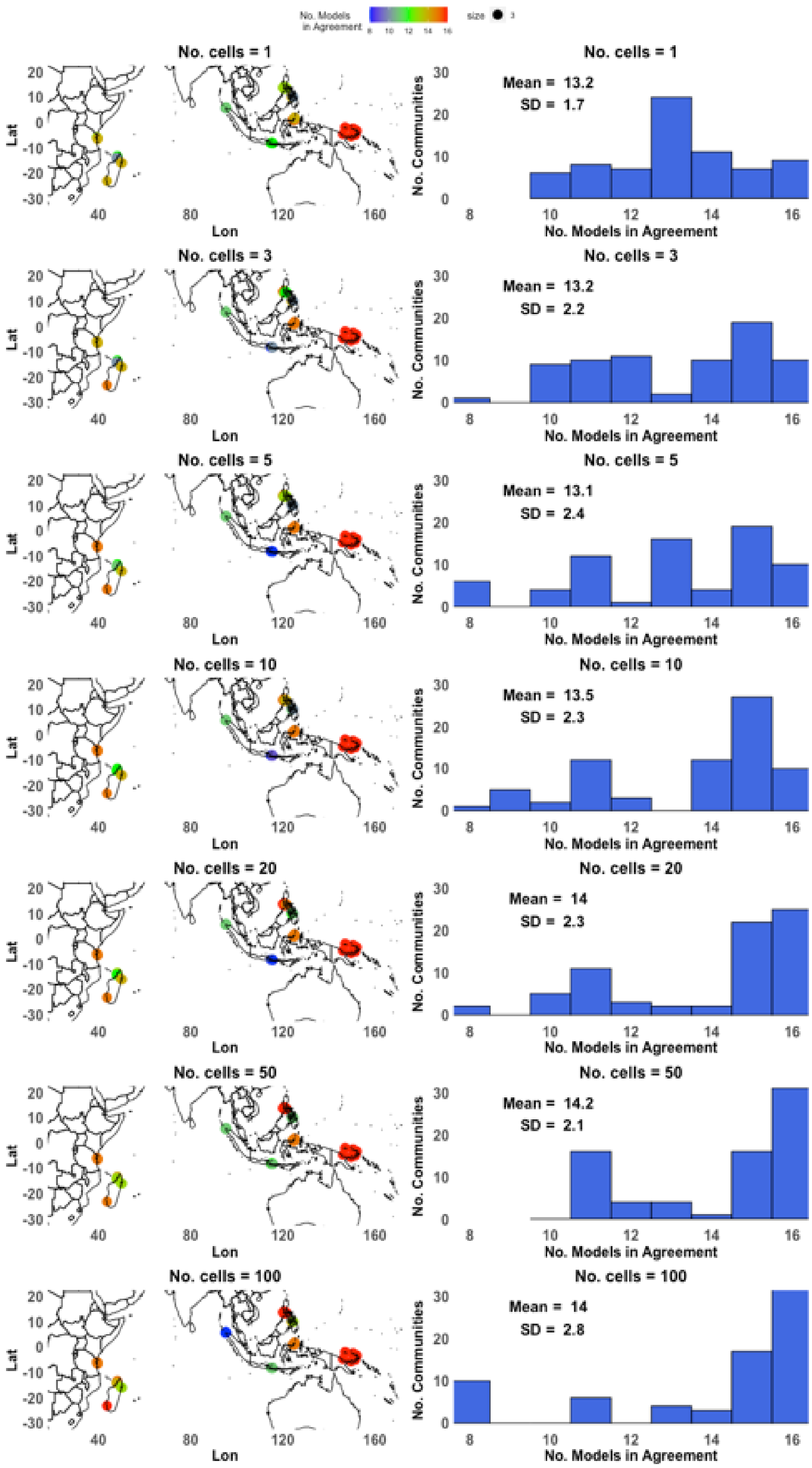

Supplementary Figure 6. **Trade-off between model agreement (colour gradient and histograms) and number of cells (rows) used for fisheries SSP1-2.6**. A model run agreement of 8, the lowest possible value, means that half of model runs indicate one direction of change, and half the opposite; conversely, a value of 16 indicates that all model runs agree on the direction of change.

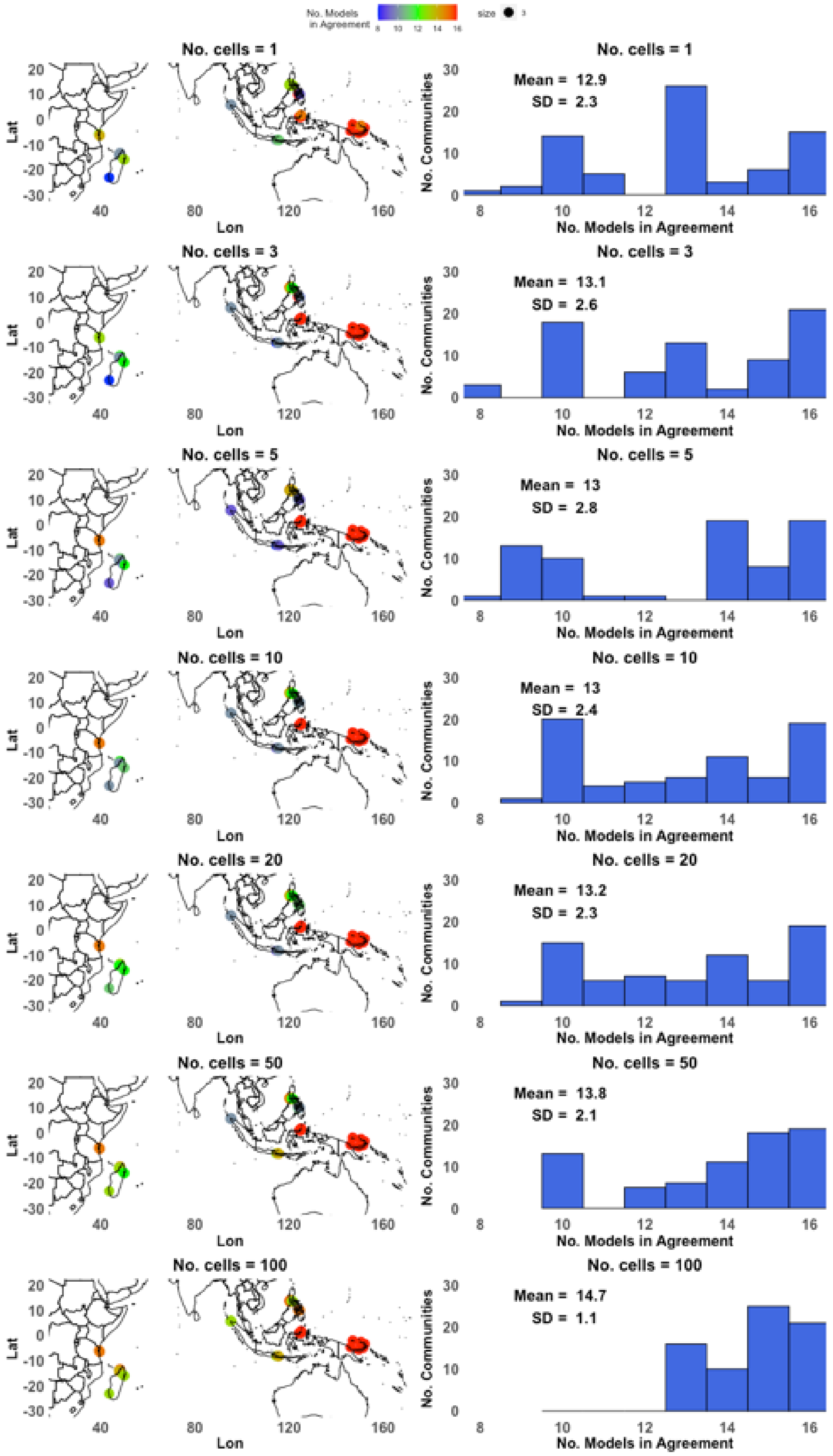

Supplementary Figure 7. **Trade-off between model agreement (colour gradient and histograms) and number of cells (rows) used for fisheries SSP5-8.5**. A model run agreement of 8, the lowest possible value, means that half of model runs indicate one direction of change, and half the opposite; conversely, a value of 16 indicates that all model runs agree on the direction of change.

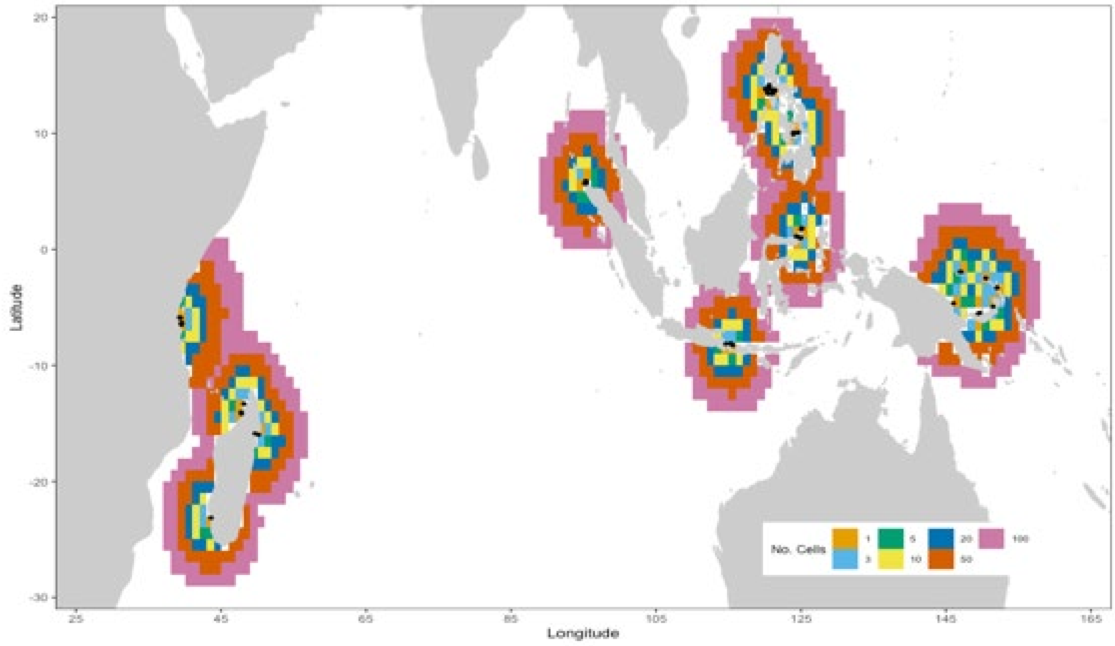

Supplementary Figure 8. **Spatial extent covered by using different numbers of grid cells (indicated by colour) to determine fisheries exposure**. Black points are study site locations.

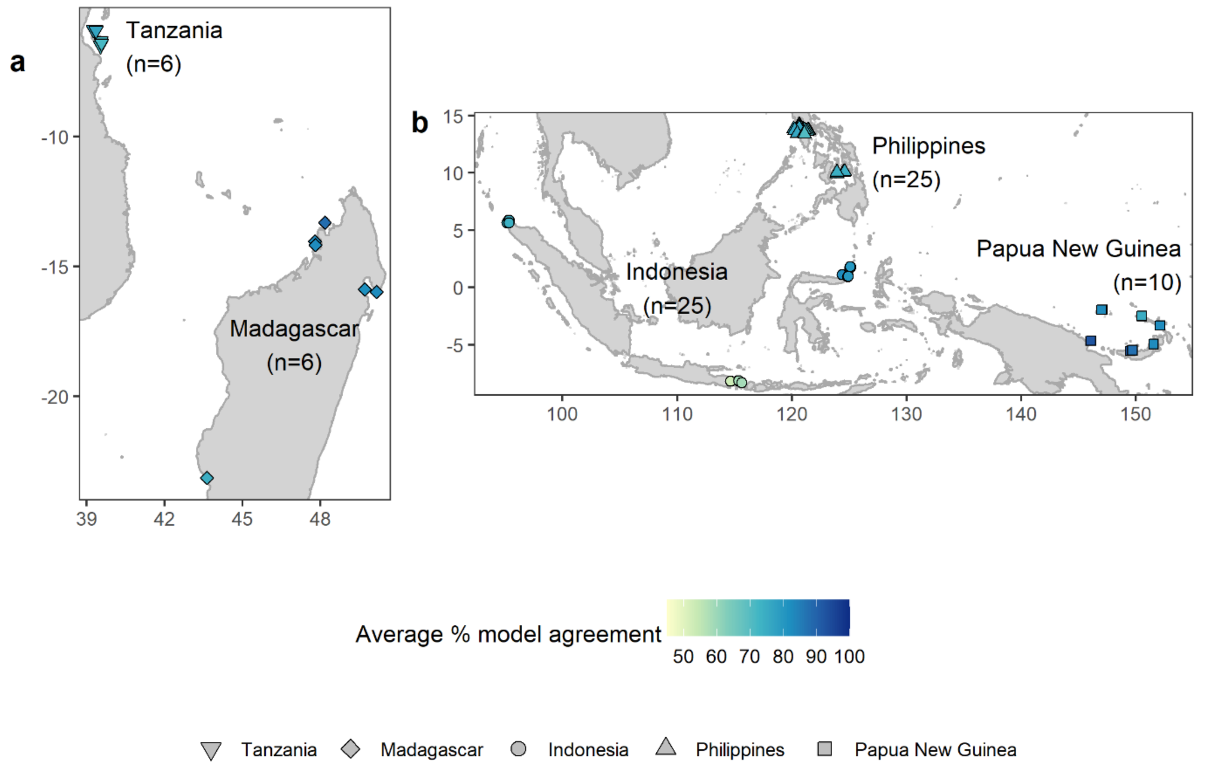

Supplementary Figure 9. **Inset map of study sites (with different shapes for each country) and average (mean) agriculture-fisheries model run agreement (indicated by colour) at each study site (n = 72)**. **a** Study sites in Tanzania and Madagascar. **b** Study sites in Indonesia, Philippines, and Papua New Guinea. A model run agreement of 50% means that half of model runs indicate one direction of change, and half the opposite; conversely, a value of 100% indicates that all model runs agree on the direction of change. Base layer map data (10m land, small islands, and coastlines) are from Natural Earth (freely available at naturalearthdata.com).
