# Supplementary material for: Potential impacts of climate change on agriculture and fisheries production in 72 tropical coastal communities
Source: Nat Commun. 2022 Jul 5;13:3530. doi: 10.1038/s41467-022-30991-4 (PMC9256605; doi:10.1038/s41467-022-30991-4)
Supplement: Supplementary file 3 — Description of Additional Supplementary Files [file 41467_2022_30991_MOESM3_ESM.pdf]

SUPPLEMENTARY DATA DESCRIPTION

File Name: Cinneretal_AgFish_SupplementaryData.csv
Description: Sample sizes and proportions of each coastal community included in social surveys
